# Modeling the Filler Phase Interaction with Solidification Front in Al(TiC) Composite Produced by the In Situ Method

**DOI:** 10.3390/ma14247560

**Published:** 2021-12-09

**Authors:** Dorota Kalisz, Paweł L. Żak, Olena Dan

**Affiliations:** Faculty of Foundry Engineering, AGH University of Science and Technology, Al. A. Mickiewicza 30, 30-059 Krakow, Poland; pawelzak@agh.edu.pl (P.L.Ż.); danelena.leo@gmail.com (O.D.)

**Keywords:** aluminum composite, filler phase interaction, flow gradient, solidification front, TiC nanoparticles

## Abstract

This paper presents simulation results of the interaction of TiC nanoparticle in liquid aluminum. The behavior of the TiC particle in the frontal interaction region stems from the operation of a system of such forces as gravity, viscous flow drag force, and Saffman force. The difference in density between the TiC and the aluminum matrix makes the particle fall, regardless of the radius dimension; while the Saffman force—which accounts for the local velocity gradient of the liquid aluminum—causes that particles with the smallest radii considered in the calculations 6.4 × 10^−8^ m; 7 × 10^−8^ m; 7.75 × 10^−8^ m; 9.85 × 10^−8^ m are repelled from the solidification front and the particles with 15.03 × 10^−8^ m are attracted to it. The viscosity growth in the course of casting caused by the lowering temperature reduces this effect, though the trend is maintained. The degree to which the particle is attracted to the front clearly depends on the velocity gradient of the liquid phase. For a very small gradient of 0.00001 m/s, the particle is at its closest position relative to the front.

## 1. Introduction

Aluminum and aluminum-based alloys are widely used in industry and everyday life for their physical and mechanical properties, wear resistance, and corrosion resistance. At the same time, obviously they cannot meet a number of requirements imposed on materials intended for the production of machine parts and mechanisms for aviation, transportation, military and biomedical industries, traditional alloys. The solution to this problem is to develop and study aluminum-based composites [1,2,3,4].

Currently, aluminum matrix composites are being used as radiation protection materials. A new class of lightweight materials based on aluminum–magnesium alloy with various ceramic fillers has also been developed and used depending on the type of ionizing radiation. Structured metallic materials have high mechanical properties but undergo significant swelling, including structural changes, when operating in the enhanced ionizing radiation mode. These structural changes can be prevented by using metals that are less susceptible to swelling and modification with various nanostructural fillers [5].

The composite materials are commonly assumed to contain two or more phases (matrix and reinforcing particles), significantly differing in properties and having a distinct interface [6]. The properties of the composite material are superior to those of its constituents. Highly plastic metal matrices, for example, aluminum alloys and high-strength fillers with high modulus of elasticity are artificially combined into a composite material. With this combination of phases, it is possible to obtain composites with the required strength, elastic modulus, microhardness and fracture toughness, and heat resistance; as well as proper dielectric, magnetic, radioactive parameters, etc. [7].

When obtaining composite materials, special attention should be paid to the selection of both the matrix alloy and its reinforcing particles. The most promising for enhancing the high-temperature strength and thermal stability of aluminum are soluble phases that are liquid-soluble and which do not contain metallic solvents (borides and carbides) or complex heat-resistant phases with high electrical conductivity. The ingredients for the synthesis of such phases in liquid aluminum are transition metals and some non-metallic elements—e.g., boron and carbon—which have a higher chemical affinity for transition metals than for aluminum.

One group of aluminum matrix composites constitute materials reinforced with titanium carbide TiC particles. According to Maziarz, Olejnik, et al. [8,9,10], an effective method to create TiC-reinforced aluminum-based composite materials is the in situ method. The chemical reactions in the in situ process in the alloy are evoked by the introduction of reactive metals, non-metals, gases or compounds and result in the formation of a thermodynamically stable reinforcing phase that is wetted by the metal due to the appearance of coherent boundaries; at the same time these compounds are thermally stable at elevated temperatures. This process takes place at high temperatures and is characterized by hardly controllable high reaction rates. Furthermore, the size of the synthesized filler phase compounds is also difficult to control in the nanoscale, and the in situ method can only synthesize a limited number of fillers.

When the aluminum infiltrated preform is heated from about 890 to 990 K, an exothermic reaction takes place [11]

Ti + 3Al = TiAl_3_ (1)

The synthesis of TiC particles occurs at about 1150 K according to the reaction

3TiAl_3_ + Al_4_C_3_ = 3TiC + 13Al (2)

Reaction (3) occurs up to about 1265 K

TiAl_3_ + C = TiC + 3Al (3)

The possibility of the synthesis to form second-phase particles in aluminum alloys can be evaluated by the size of the thermodynamic potential of the reaction of their formation (Table 1) [12].

The analysis of the relationships ΔG°T = f (T) and log K = f (T) shows that the studied phases can be synthesized in a wide range of temperatures. In the Al-C-Ti system, the reactions of titanium carbide (TiC) formation are the most favorable; at temperatures above 1000 K, the possibility of aluminum carbide formation is excluded. The presence of Al4C3 would be negative as it is hydrophilic and reduces the adhesive bond between the filler and matrix. Up to temperature 900–950 °C aluminum does not wet titanium carbides, and at temperatures above 1000–1050 °C the contact angle rapidly decreases to zero. This phenomenon is related to the sublimation of the alumina layer on the droplet surface and the intensification of chemical reactions at the interface between the aluminum and solid phase components [12].

In addition to above-mentioned thermodynamic factors and physicochemical phenomena, the final properties of the composite and its suitability will be affected by physicochemical phenomena occurring during solidification, involving flotation processes and interactions with the solidification front of the filler particles (TiC) of the composite. The solidification process is accompanied by the segregation of alloy components, precipitation processes, and interaction of the formed particles with the solidification front. Consequently, the particles may be uniformly dispersed in the cast composite or form clusters This phenomenon will be determined by repulsion and absorption processes, which in turn will depend on, among other things, the size and density of the particle and alloy and wettability. In the case of nanoparticles produced by the in situ method in composites, the experimental studies focused on the formation of the TiC phase through the introduction of an appropriate amount of the moderating powder; then the microstructure and chemical composition of the cast composites were studied using a LeicaDM IRM light microscope (LM), a FEI ESEM XL30 scanning electron microscope (SEM) equipped with an X-ray energy dispersive spectrometer. For nanoparticle phase reinforced composites, it is important to understand the mechanisms shaping their microstructure and nanoparticle phase distribution. As for the traditional studies based on melting and then observing samples, no observation of processes occurring at the liquid–solid interface of composites is possible. This, however, can be done with the computer program presented in this paper, used for simulating the process of interaction of the formed TiC nanoparticles with the solidification front.

## 2. Materials and Methods

The tests were carried out for a composite material made on the matrix of the aluminum 1000 casting alloy, the chemical composition of which was determined using a JY 10,000 RF glow discharge spectrometer (IMiIM PAN, Cracow, Poland), as shown in Table 2 [8,10].

**Figure 1:**
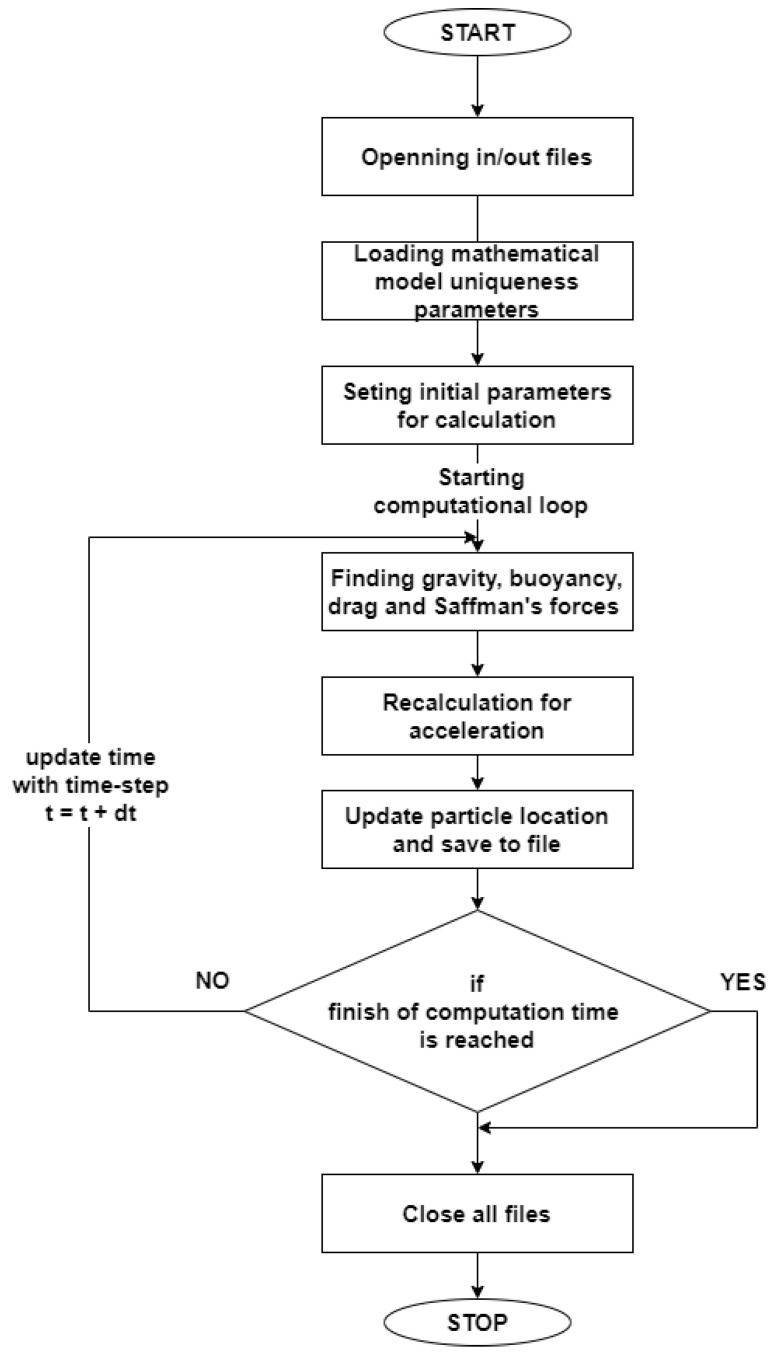
Block diagram of the computer program.

The composite material was produced by in situ casting by self-propagating high-temperature synthesis in bath (TiC). A mixture of aluminum powders was used in the process as moderator and titanium and graphite as TiC forming reactants. The purity of the powders was at least 99.9%. The amount of TiC in the composite correspond to about 5 mass %. Studies on the phenomenon of TiC filler phase particles interaction with the solidification front were performed using a computer program written for the needs of this work. The block diagram is shown in Figure 1. Calculations were made for the flat front and ideal solidification. During computation process time domain was divided into short parts (time steps). At each of the time step change of the related forces and precipitation acceleration change is so small and is assumed to be close to zero. Spatial domain is assumed to be continuous. In order to simplify the computational process, calculations took place in 2D domain. Computational loop covers fallowing stages: finding values of the forces that governs the process, calculation of the acceleration on the base of resultant force, acceleration allows to calculate temporary velocity and in effect new position of the particle may be found. The resultant force at each time step is calculated as resultant vector on the base of gravity, buoyancy, drag, and Saffman’s forces which are vectors. Adding and subtracting vectors is realized with use of trigonometric functions for the triangles that define parallelograms. Similarly, acceleration computed on the basis of resultant force is then divided into component vectors, which are parallel to the *x*-and *y*-axes. This allows to calculate movement by two equations that describe precipitation velocity in the *x*- and *y*-axis. Similarly, the location vector can be found on the base of two suitable equations. After update of the location, the process time value is also updated and next loop starts from the beginning. Computations are stopped when finish time value is reached. The finish time value is input data for computation.

During solidification, particles can be absorbed or repelled by the moving front. These interactions have an effect in the degree of particle dispersion, and it should be assumed that clustering in certain areas of the composite is also possible. Depending on the chemical composition of the filling phase—including density, radius, and degree of wettability by the liquid metal—the behavior of the particle with respect to the moving solidification front may vary. Most of the information on the nature of the phenomenon comes from studies on model transparent liquids with low melting points, in which particle motion was observed for sizes 5–1000 μm. The simplest criterion for determining whether absorption of a particle by a solidification front takes place is the magnitude of the free energy of absorption ∆G_p_, defined as the phase-to-phase energy difference σ_p-s_ (particle—solid metal) and σ_p-l_ (particle—liquid metal). Absorption occurs when [14,15]

∆G_p_ = σ_p-s_ − σ_p-l_ ≤ 0 (4)

where:

u_p-s_—surface energy of particle—solid metal system (N/m),

σ_p-l_—surface energy of particle—liquid metal system (N/m),

∆G_p_—free energy of particle absorption.

This condition applies for low rates of displacement of the solidification front.

**Figure 2:**
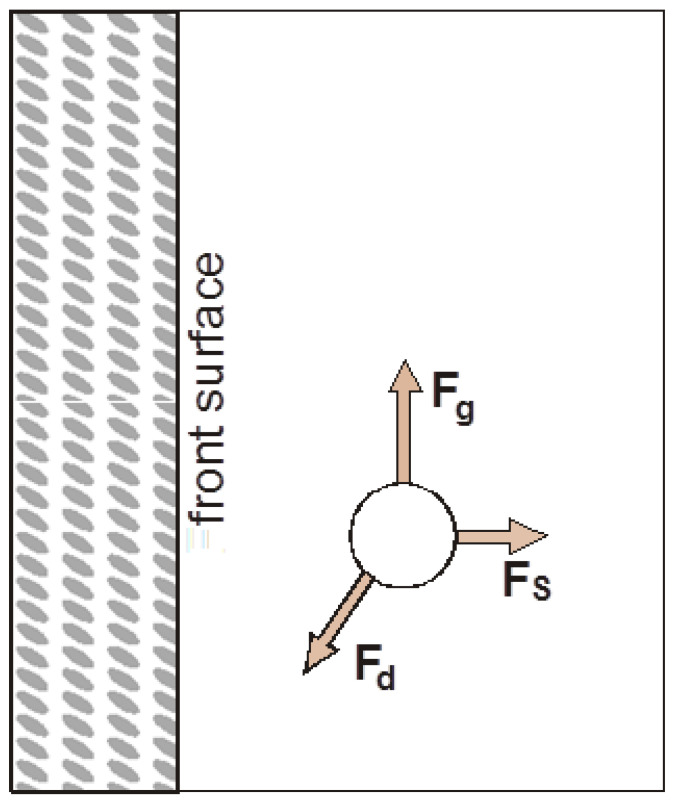
System of forces acting on a particle in the area of influence of the solidification front.

The simplest case is the interaction of a single particle in the vicinity of a crystallization front in a system where there is no convective fluid flow. If the particle is in the area of interaction of a horizontally moving solidification front, it will move with respect to the front and with respect to the surrounding liquid phase [15,16,17,18]. The motion of the particle will be evoked by a system of forces acting on it: gravitational force, viscous flow resistance force (acting opposite to the motion of the particle), Saffman force (Figure 2) [19,20].

Gravitational force (i.e., the difference between buoyancy and gravity)

(5) $$F_{g}=\frac{4}{3}\times \pi \times r^{3}\left(\rho_{m}-\rho_{p}\right)\times g$$

where:

*F_g_*—difference of buoyancy force and gravity (N), *ρ_m_*—density for a particlle distant from the frontu of metal (steel) density (kg m^−3^), *ρ_p_*—particle (inclusions) density (kg m^−3^), *g*—gravitational acceleration (m s^−2^), *r*—radius of a non-metallic inclusion (m).

Flow drag force

(6) $$F_{d}=6\times \pi \times \mu \times r\times V_{p}\times \theta$$

where:

*F*_d_—drag force of viscous flow; *µ*—dynamic viscosity (kg (m·s)^−1^); *V_p_*—particle speed (length of velocity vector) relative to fluid (m s^−1^).

The value of coefficient *θ* depends on the direction of motion of the particle and its distance from front *h*; for a particle far from the front: *θ* = 1, for a particle travelling in the direction of the front

(7) $$\theta =\frac{r}{h}$$

and for a particle moving parallel to the front

(8) $$\theta =\ln\left(\frac{r}{h}\right)$$

Near the solidification front the fluid velocity is non-homogenous (the component parallel to the front is important). 

The Saffman force is evoked by the liquid velocity gradient *S* in the direction perpendicular to the surface of the front, occurring in the region that can be identified as the velocity boundary layer, is called the Saffman force [21]. The Saffman force is defined by

(9) $$F_{s}=6.46\times \mu \times r^{2}\times V_{p}\times \sqrt{\frac{S}{v}}$$

where:

*F_s_*—Saffmann force (N), *r*—radius of a precipitate particle (m), *ν*—kinematic viscosity (m^2^ s), *S*—local fluid velocity gradient (m s^−1^).

This force can make the particle move closer or further away from the front depending on the direction of fluid movement and the density difference between the particle and the liquid metal. With the liquid flowing down along the front and for a particle with a higher density than the liquid metal, the Saffman force moves the particle away from the front [22].

The fluid velocity gradient in the vicinity of the solidification front can be approximated by the Equation (10).

If the particle is not attracted or repelled from the front, the system of forces acting on the particle balances

(10) $${\bar{F}}_{g}+{\bar{F}}_{S}+{\bar{F}}_{d}=0$$

By substituting the equations for the individual forces into Equation (10), the so-called critical value of the solidification front displacement can be determined. If the solidification rate is lower than the critical rate, only particle repulsion will take place, with higher absorption. The smaller the filler particle size, the higher the critical solidification rate.

During solidification, the behavior of the particles in the front interaction area will be influenced by the flow and movement of the liquid metal. This flow can inhibit the absorption of particles by the solidification front. In order to simplify this model, the assumption that the movement only in *z* axis both for liquid and particle may be introduced. The particle initial velocity relative to the liquid metal, can be determined from the buoyancy and will be in the form

(11) $$v_{0}=\frac{2r^{2}\times g}{9\mu }\times \left(\rho_{p}-\rho_{m}\right)$$

The fluid velocity gradient near the solidification front can be determined from Equation (12)

(12) $$S=\frac{4\times V_{0}}{h}.$$

where: *V*_0_ denotes liquid down-flow speed parallel to *z* axis.

During the process position of the particle in *z* axis will be denoted as *z* in the further equations. Thus, the velocity of the particle, $u_{p}$, relative to the liquid matrix of the vertical direction, can be determined from the relation

(13) $$u_{p}=v_{0}\times \left(1-\frac{{\left(h-z\right)}^{2}}{h^{2}}\right)$$

where *z* denotes horizontal coordinate.

Then the Saffman force can be determined as

(14) $$F_{S}=6.46\times \mu \times r^{2}\times \left(\frac{4\times V_{0}}{h\times \nu }\right)\times v_{0}\times \left(1-\frac{{\left(h-z\right)}^{2}}{h^{2}}\right)$$

which is force that cause particle movement in the direction parallel to the vertical crystallization front.

## 3. Results and Discussion

**Figure 3:**
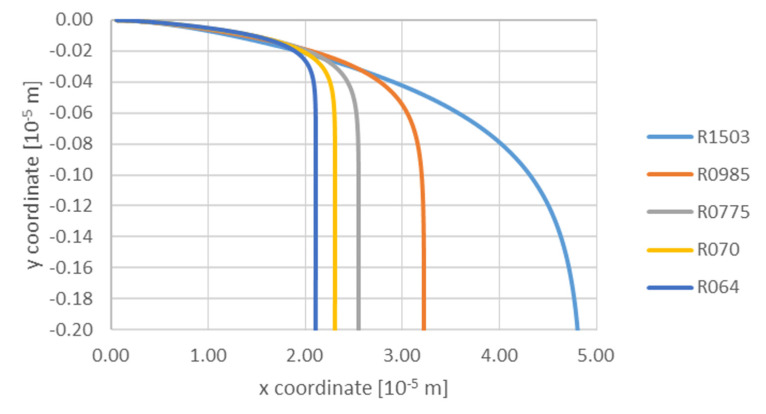
Analysis of influence of TiC particle size on the trajectory at a constant gradient 0.00001 m/s and viscosity 0.0013 Pa·s: R064—6.40 × 10^−8^ m; R070—7.00 × 10^−8^ m; R0775—7.75 × 10^−8^ m; R0985—9.85 × 10^−8^ m; R1503—15.03 × 10^−8^ m.

**Figure 4:**
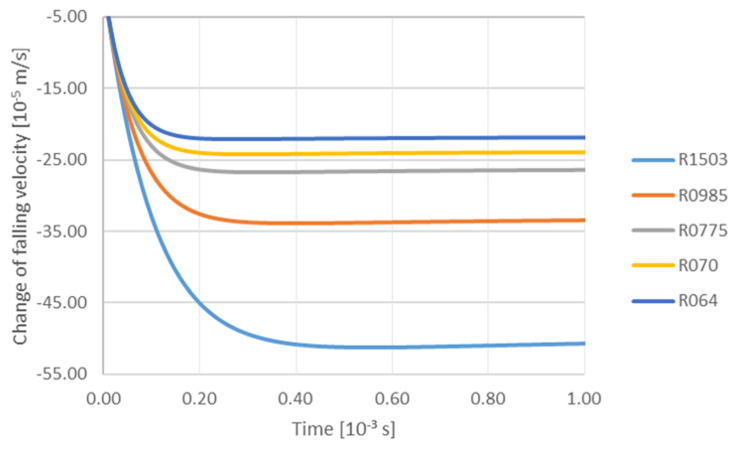
Analysis of influence of TiC particle size on the change of falling velocity depending on time with a constant gradient 0.00001 m/s and viscosity 0.0013 Pa·s: R064—6.40 × 10^−8^ m; R070—7.00 × 10^−8^ m; R0775—7.75 × 10^−8^ m; R0985—9.85 × 10^−8^ m; R1503—15.03 × 10^−8^ m.

The following parameters were assumed in the calculations: flow gradient (liquid phase) G 0.000001–0.01 m·s^−1^, particle radius TiC 6.4 × 10^−8^ m; 7 × 10^−8^ m; 7.75 × 10^−8^ m; 9.85 × 10^−8^ m; 15.03 × 10^−8^ m; and viscosity of aluminum 0.0013–0.002 Pa·s. The simulation results are presented in the successive figures. Figure 3 shows the effect of TiC particle size on its trajectory at a constant velocity gradient (liquid Al) 0.00001 m·s^−1^, in the area of influence of the solidification front. It can be seen from the trajectories of particles of the investigated size group that all they fall down, which is a consequence of the density difference between the TiC particle and the Al matrix. As observed at the initial stage of the process, the particles are attracted by the front up to a distance of ca. 1.8 × 10^−5^ m, while particles with the smallest radii fall, remaining at a constant distance from the front. The attraction mechanism continues to act on particles with radii 9.85 × 10^−8^ m; 15.03 × 10^−8^ m, with the attraction mechanism acting most intensively on particles with the largest radius. The mechanism of particle descent is shown in Figure 4 in the initial period, i.e., up to ca. 0.05 × 10^−3^ s particles fall with equal speed—after which the falling effect is mainly due to the density difference between the particle and the matrix. Accordingly, the particles with radius 15.03 × 10^−8^ m fall fastest. Observing the curves of the particle’s trajectory one notices that all purges decelerate at a certain distance. This is due to the fact that the force associated with gravity is balanced by the force associated with the viscous flow resistance. The particle deceleration occurs first for the smallest particles (Figure 3).

**Figure 5:**
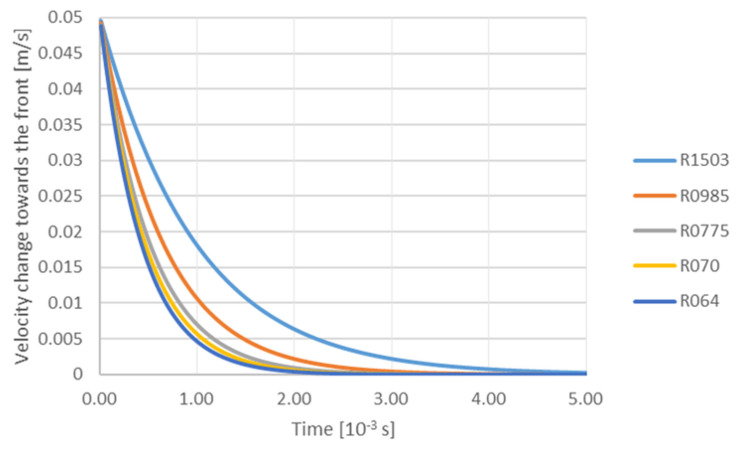
Analysis of influence of TiC particle size on the velocity change towards the front depending on time with a constant gradient 0.00001 m/s and viscosity 0.0013 Pa·s: R064—6.40 × 10^−8^ m; R070—7.00 × 10^−8^ m; R0775—7.75 × 10^−8^ m; R0985—9.85 × 10^−8^ m; R1503—15.03 × 10^−8^ m.

The next figure (Figure 5) shows the effect of TiC particle size on the change in velocity in the front direction as a function of time at a constant gradient of 0.00001 m/s and viscosity 0.0013 Pa·s: R064—6.40 × 10^−8^ m; R070—7.00 × 10^−8^ m; R0775—7.75 × 10^−8^ m; R0985—9.85 × 10^−8^ m; R1503—15.03 × 10^−8^ m. The initial velocity of all TiC particles towards the front was assumed to be 0.05 m/s. The resulting trajectory of individual particles shows that all particles decelerate. The TiC particles with radii of 0.064; 0.070 and 0.0775 m completely slow down after 2 × 10^−3^ s, whereas for particles with a radius of 15.03 × 10^−8^ m this phenomenon occurs after 4.0 × 10^−3^ s.

**Figure 6:**
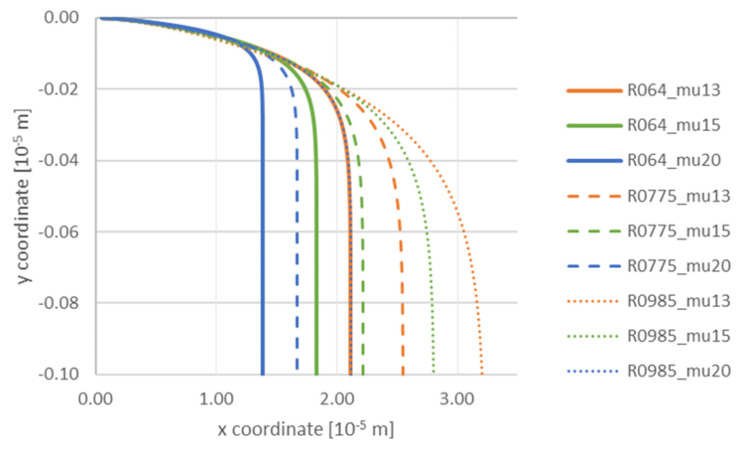
Analysis of influence of TiC particle size and viscosity on the trajectory with a constant gradient 0.00001 m/s. Radius of particle: R064—6.40 × 10^−8^ m; R0775—7.75 × 10^−8^ m; R0985—9.85 × 10^−8^ m. Viscosity: mu13—0.0013 Pa·s; mu15—0.0015 Pa·s; mu20—0.0020 Pa·s.

Next, the influence of particle size and viscosity of liquid aluminum on the motion at a constant gradient of 0.00001 m/s was analyzed (Figure 6).

**Figure 7:**
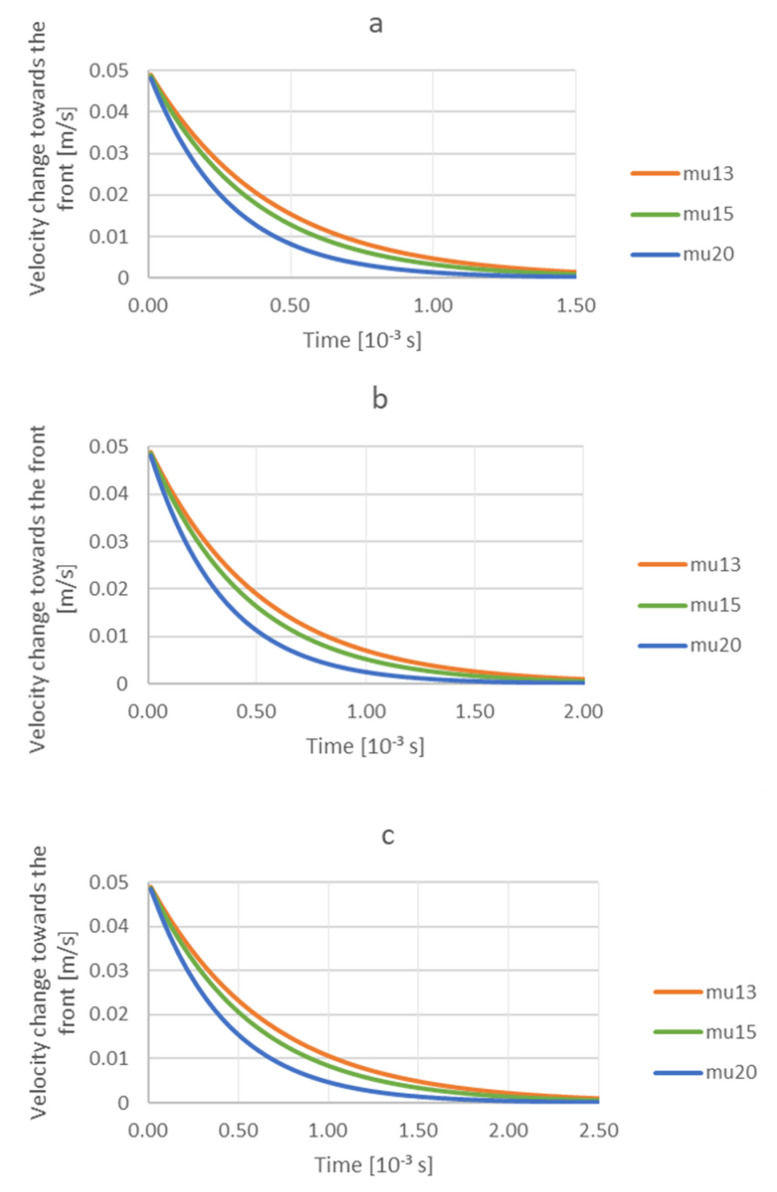
Analysis of the influence of TiC particle size and viscosity on the velocity change towards the front as a function of time with a constant gradient 0.00001 m/s. Radius of particle: (**a**) 6.40 × 10^−8^ m; (**b**) 7.75 × 10^−8^ m; (**c**) 9.85 × 10^−8^ m. Viscosity: mu13—0.0013 Pa·s; mu15—0.0015 Pa·s; mu20—0.0020 Pa·s.

The analysis of the trajectory of the particle in the region of the front reveals that all TiC particles fall down. This tendency is similar to the one presented in Figure 3. Particles with a small radius are repelled from the front. The increasing or decreasing viscosity does not change this tendency, but it intensifies it when higher viscosity of aluminum is involved. This means that during solidification by means of lowered temperature, the viscosity of the liquid medium increases, causing that particles with a very small radius are more readily repelled from the front. Similarly, in the case of particles with radius 7.75 × 10^−8^ m and 9.85 × 10^−8^ m, the particles are placed further from the front when the viscosity grows. At the same time, it is evident from the trajectory of TiC particles with these radii that they are attracted by the front, and this phenomenon is more intense for low values of viscosity (0.0013 Pa·s). The following figures (Figure 7a–c) present simulation results and the effect of TiC particle size and viscosity on the variation of TiC particle velocity toward the front versus time at a constant gradient of 0.00001 m/s. Particle radius: a—6.40 × 10^−8^ m; b—7.75 × 10^−8^ m; c—9.85 × 10^−8^ m. Viscosity: mu13—0.0013 Pa·s; mu15—0.0015 Pa·s; mu20—0.0020 Pa·s. The same trend was observed in all three analyzed cases. It resulted in a decrease of the particle velocity due to the balancing of the gravitational force by the drag force of the viscous flow. This process occurred earlier for higher viscosities and small radius particles (Figure 7a).

**Figure 8:**
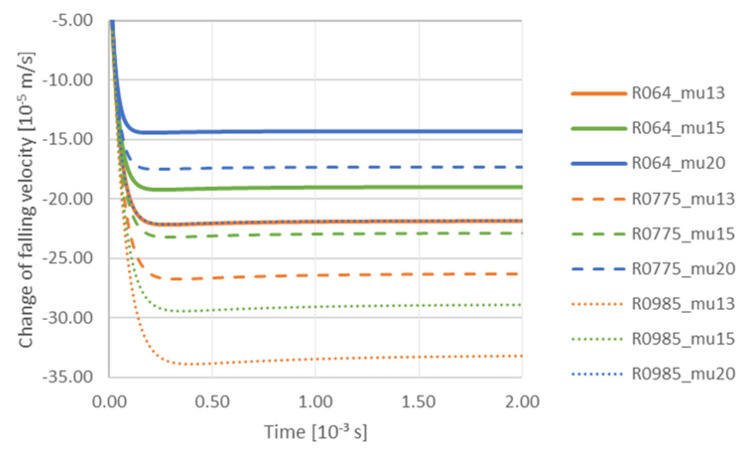
Analysis of influence of TiC particle size and viscosity on the change of falling velocity with time at a constant gradient 0.00001 m/s. Radius of particle: R064—6.40 × 10^−8^ m; R0775—7.75 × 10^−8^ m; R0985—9.85 × 10^−8^ m. Viscosity: mu13—0.0013 Pa·s; mu15—0.0015 Pa·s; mu20—0.0020 Pa·s.

**Figure 9:**
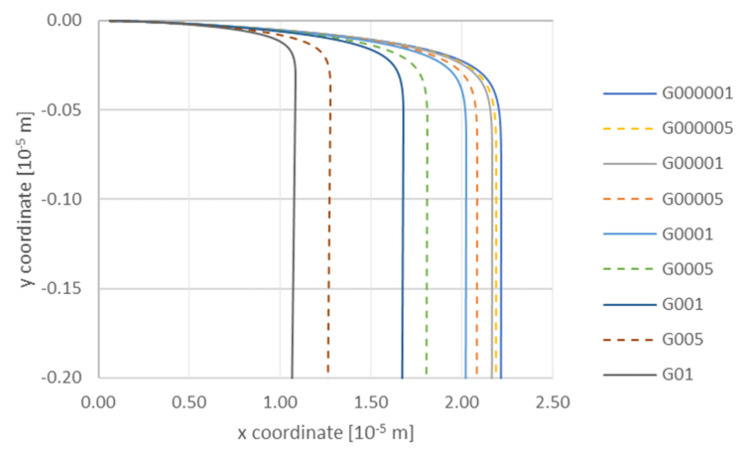
Analysis of influence of gradient on the trajectory of a TiC particle with a radius 7.75 × 10^−8^ m under the conditions of viscosity 0.0015 Pa·s. Gradient, m/s: G000001—0.00001; G000005—0.00005; G00001—0.0001; G00005—0.0005; G0001—0.001; G0005—0.005; G001—0.01; G005—0.05; G01—0.1.

**Figure 10:**
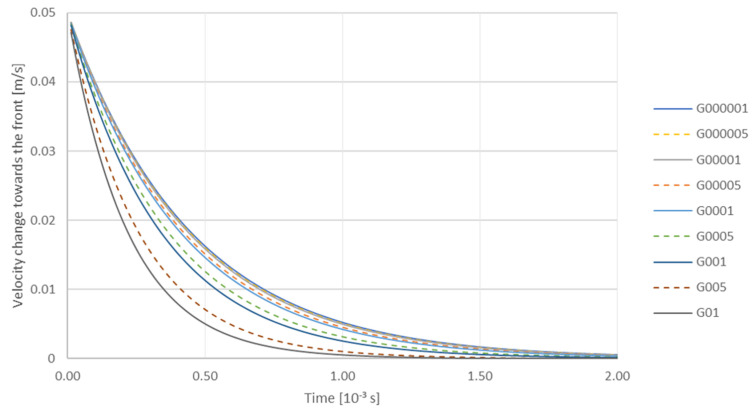
Analysis of influence of gradient on the change of velocity of a TiC particle with a radius of 7.75 × 10^−8^ m towards the front under the viscosity conditions of 0.0015 Pa·s. Gradient, m/s: G000001—0.00001; G000005—0.00005; G00001—0.0001; G00005—0.0005; G0001—0.001; G0005—0.005; G001—0.01; G005—0.05; G01—0.1.

At the next stage, authors analyzed the influence of TiC particle size and viscosity on the variation of the falling velocity with time at a constant gradient of 0.00001 m/s. Particle radius: R064—6.40 × 10^−8^ m; R0775—7.75 × 10^−8^ m; R0985—9.85 × 10^−8^ m. Viscosity: mu13—0.0013 Pa·s; mu15—0.0015 Pa·s; mu20—0.0020 Pa·s (Figure 8). The increase in viscosity causes deceleration of TiC particles, and the particles with the smallest radius of 6.40 × 10^−8^ m slow down first. On the other hand, the analysis of the effect of the liquid phase velocity gradient on the trajectory of a TiC particle—with radius 7.75 × 10^−8^ m when the viscosity equals to 0.0015 Pa·s (Figure 9)—showed that the gradient had a significant impact on the interaction of the particle with the solidification front. At a high gradient of the liquid phase velocity the particle motion path was observed to run at a considerable distance from the front (for a gradient of 0.01 m/s the particle was attracted to the front at a distance 1 × 10^−5^ m). Then it descended, remaining at a constant distance from the front. The tendency of the particle to be attracted to a certain distance from the front was observed for all velocity gradients considered here, and it was also characteristic that the particle was falling at a constant distance from the front. The degree of attraction of the particle to the front clearly depended on the velocity gradient of the liquid phase. At a very small gradient of 0.00001 m/s, the particle was closest to the front. On the other hand, the analysis of the influence of the velocity gradient on the change of the TiC particle velocity towards the front with radius 7.75 × 10^−8^ m showed that in this case the increase of liquid phase velocity gradient influenced the decrease of TiC particle velocity (Figure 10).

**Figure 11:**
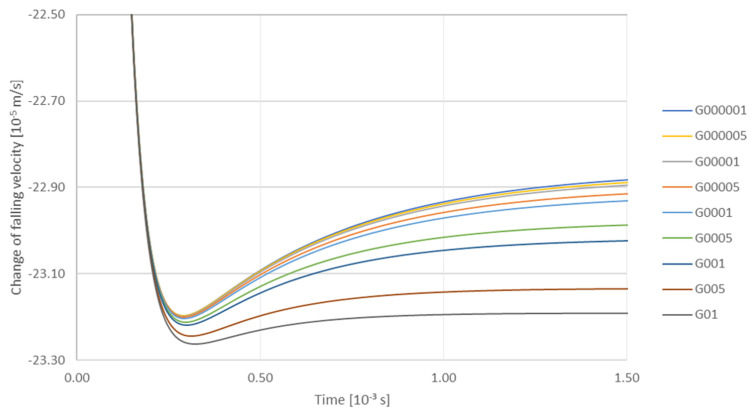
Analysis of the influence of gradient on the change of falling velocity of a TiC particle with a radius of 7.75 × 10^−8^ m under the viscosity conditions of 0.0015 Pa·s. Gradient, m/s: G000001—0.00001; G000005—0.00005; G00001—0.0001; G00005—0.0005; G0001—0.001; G0005—0.005; G001—0.01; G005—0.05; G01—0.1.

Then the influence of the gradient on the change in the falling velocity of a TiC particle with radius 7.75 × 10^−8^ m was analyzed for: Viscosity—0.0015 Pa·s. Gradient, m/s: G000001—0.00001; G000005—0.00005; G00001—0.0001; G00005—0.0005; G0001—0.001; G0005—0.005; G001—0.01; G005—0.05; G01—0.1 (Figure 11). It can be seen that the trajctories of particles during their descent coincide with one another. At the initial stage, i.e., up to ca. 0.25 × 10^−3^ s particles fall down to a minimum velocity, then after a time of approx. 0.5 × 10^−3^ s the minimum speed increases, which is shown in the figure as a characteristic elevation of the trajectory from 0.5 × 10^−3^ s to 1 × 10^−3^ s (Figure 11). On the other hand, the analysis of influence of the gradient on the change of the velocity vector length indicates that for a particle with radius 7.75 × 10^−8^ m, when the viscosity equals to 0.0015 Pa·s, Gradient, m/s: G000001—0.00001; G000005—0.00005; G00001—0.0001; G00005—0.0005; G0001—0.001; G0005—0.005; G001—0.01; G005—0.05; G01—0.1 the velocity gradient makes the length of the velocity vector decrease, with the increase of the gradient being the most intense factor (Figure 11).

## 4. Conclusions

The behavior of TiC nanoparticle in the interaction region of the solidification front in aluminum matrix is affected by several parameters: density difference between TiC and Al, liquid phase velocity gradient, aluminum viscosity. The analysis of the trajectory lines of TiC particles with variable radii reveals that the gravitational force causes the fall of particles of all radii; additionally, the Saffman forces acting here and the drag force cause that TiC particles of very small radius are initially attracted to the front and then repelled and remain at a constant distance from the solidification front. This effect is increased for liquid aluminum with low viscosity. The acting system of forces associated with viscous flow resistance and gravity also makes the particle velocity decrease, and this effect is strongest for TiC particles with the smallest radius. The motion path of the TiC particle is affected by the velocity gradient of the liquid phase; increasing this magnitude causes a gradual slowing down of the TiC particle motion.

## Figures and Tables

**Table 1:** Temperature dependence of Gibbs energy and equilibrium constants of the formation reaction of some phases in liquid aluminum [12,13].

Reaction S—Solid, l—Liquid	ΔG°T, kJ/mol	lgK
Ti_s_ + C_s_ = TiC_s_	−184.889 + 0.0125 T	9652/T—0.655
4Al_l_ + 3C_st_ = Al_4_C_3s_	−265.150 + 0.095 T	13,842/T—4.960
3Al_l_ + Ti = TiAl_3s_	−37.5 + 0.0106 T	1958/T—0.553

**Table 2:** Chemical composition of composite matrix—Al 1000 alloy (mass %).

Al (min.)	Contaminants (max.)					
	Fe	Si	Cu	Zn	Ti	Total
99.7	0.2	0.15	0.01	0.04	0.01	0.3

## Data Availability

Data are contained within the article.
